# OpenDrop: An Integrated Do-It-Yourself Platform for Personal Use of Biochips

**DOI:** 10.3390/bioengineering4020045

**Published:** 2017-05-19

**Authors:** Mirela Alistar, Urs Gaudenz

**Affiliations:** Gaudi Labs, Lucern 6003, Switzerland; info@gaudi.ch

**Keywords:** droplet microfluidics, lab-on-a chip, electromicrofluidics, design automation, open source hardware, do-it-yourself biology

## Abstract

Biochips, or digital labs-on-chip, are developed with the purpose of being used by laboratory technicians or biologists in laboratories or clinics. In this article, we expand this vision with the goal of enabling everyone, regardless of their expertise, to use biochips for their own personal purposes. We developed OpenDrop, an integrated electromicrofluidic platform that allows users to develop and program their own bio-applications. We address the main challenges that users may encounter: accessibility, bio-protocol design and interaction with microfluidics. OpenDrop consists of a do-it-yourself biochip, an automated software tool with visual interface and a detailed technique for at-home operations of microfluidics. We report on two years of use of OpenDrop, released as an open-source platform. Our platform attracted a highly diverse user base with participants originating from maker communities, academia and industry. Our findings show that 47% of attempts to replicate OpenDrop were successful, the main challenge remaining the assembly of the device. In terms of usability, the users managed to operate their platforms at home and are working on designing their own bio-applications. Our work provides a step towards a future in which everyone will be able to create microfluidic devices for their personal applications, thereby democratizing parts of health care.

## 1. Introduction

Microfluidics, the study and handling of small volumes of fluids, has the potential to revolutionize the laboratory research with immediate applications in medical care (e.g., point-of-care diagnostics [1,2,3] and drug discovery [4,5,6]). Such applications on biological materials, typically including experimental procedures, recipes and data analyses, are commonly known as “bio-protocols”. The explicit benefit of microfluidics for bio-protocols, stems from the miniaturization of fluids that leads to reduced material consumption and faster time-to-result. However, microfluidics can further benefit from automation and reconfigurability. That is why, in the past decade, microfluidics research has become increasingly multidisciplinary, involving such varied fields as nanotechnology, electrical engineering and computer science.

Microfluidic research is typically conducted in one of the following directions: (i) building the microfluidic machines that reliably manipulate fluids [7,8,9,10,11,12,13]; (ii) designing new bio-protocols for microfluidics [1,2,3,4,5,6]; or (iii) developing automation algorithms for the execution of bio-protocols on the microfluidic machines [14,15,16,17,18].

Recently, research trends have shifted towards *integrated* microfluidic platforms that provide a complete workflow from bio-protocol specification to microfluidic operations.

Microfluidic platforms can be classified according to the liquid propulsion principle used for operation, e.g., capillary, pressure driven, centrifugal, electrokinetic or acoustic.

**Figure 1:**
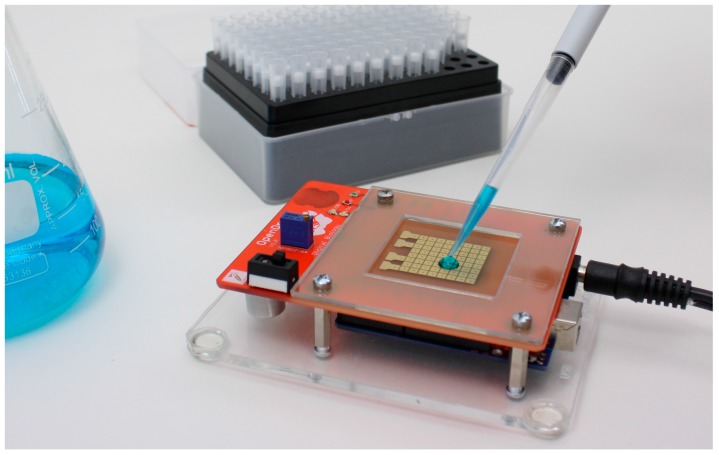
OpenDrop consists of an array of electrodes, with each electrode capable of holding a droplet. To execute bio-applications (“bio-protocols”), OpenDrop moves the micro-droplets by means of electrical voltage. OpenDrop is compact and portable as the electrode array can be actuated directly from battery with no need for additional pressure or vacuum pumps.

We are interested in *digital* microfluidic biochips, which manipulate the fluids as droplets on an array of electrodes, using electrical voltage [8]. In Figure 1 we show OpenDrop, the do-it-yourself (DIY) biochip we developed for personal use.

Digital microfluidic biochips manipulate droplets by applying electrical voltage. The phenomenon is called ‘electro-wetting on dielectric’ (EWoD) [8,9]. Electrical voltage unbalances the force equilibrium at the solid-liquid-vapor interface, causing the droplets to move towards the charged electrodes [10,11]. Using EWoD, biochips can create, transport, mix and split droplets [12].

As shown in Figure 1, OpenDrop inherits the advantages of a biochip, i.e., it is: (a) compact and portable because the electrode array can be actuated directly from battery with no need for additional pressure or vacuum pumps; (b) reconfigurable because the electrodes can be used interchangeably; and (c) programmable because the droplet movement can be programmed directly from the computer [14,15,16,17,18].

Due to their advantages, biochips have great potential of truly accomplishing the vision of ‘lab-on-a chip’, that is, a complete and automated wetlab in miniature [7,8].

## 2. Motivation and Vision

Biochips, such as Neoprep (Illumina, San Diego, CA, USA) or Dropbot [19], are developed with the purpose of being used by lab technicians or biologists in laboratories or clinics [20,21]. In this paper, we expand this vision, by exploring the possibility of developing an *integrated platform for personal use of biochips*. Such a platform will enable interested users to develop and program their own applications on biochips.

As mentioned, biochips have the advantage of “programmability”; thus, the users can define their own application. When we expand the usability of biochips from biologists to a more general user, the application area also enlarges from the intended bio-protocols to unexpected applications, ranging from fragrance design to molecular gastronomy. Thus, in this paper we prefer to use the more general term “*bio-applications*” instead of “bio-protocol”, to cover applications that deviate from the common understanding of a bio-protocol.

**Figure 2:**
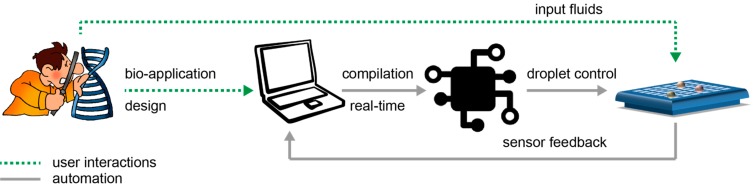
System setup. The user actions are marked with dashed lines and the automated actions are marked with solid lines. The user designs the application on the computer and then loads the microfluidic machine with the input fluids (i.e., samples, reagents, buffers). On the computer, the dedicated software compiles the application to automatically control the movement of the droplets. When the application finishes, the feedback from the sensors is recorded by the computer and transformed into data to be further read and analyzed by the user.

In Figure 2, we schematically depict the envisioned interaction between a non-expert and a biochip. First, the user designs a new bio-application on the computer. Next, the user loads the microfluidic machine with the input fluids (i.e., samples, reagents, buffers) and connects the microfluidic machine to the computer.

On the computer, the dedicated software compiles the bio-application to automatically control the movement of the fluids. When the bio-application finishes, the feedback from the sensors is recorded by the computer and transformed into data to be further read and analyzed by the user.

While we want to keep an open mind and not hinder the creativity of the users in any way, we believe there is still a significant amount of users that have enough knowledge to adapt existing bio-protocols for their own needs. Examples of bio-protocols that can be potentially customized by users are: fast assay for blood grouping [22], semen monitoring [23], bacteria detection in water [24], etc. In this paper, we focus on enabling these knowledgeable users to use biochips for developing customized bio-protocols.

We believe our work is a first step towards *personal laboratories:* small portable devices that people can own and use to develop customized bio-protocols, similar to today’s personal computers.

## 3. Our Contributions

Building a biochip for personal use encounters challenges in terms of (i) cost; (ii) accessibility; and (iii) operability. In this paper, we introduce OpenDrop, a platform that addresses all these challenges as follows.

Cost is the main concern for users. For that reason, we designed OpenDrop to be within the affordable price range for consumers. We break down the costs of OpenDrop into (a) fabrication costs: design, production and assembly costs; and (b) operation costs: the costs of the fluids used for the targeted bio-application. (a) We minimize the design cost by publishing the design files open-source, thus enabling everyone to use it directly, at no design cost. We also focused on reducing the production costs, opting to use printed-circuit-board (PCB) for the electrode substrate. When ordered online, the components of OpenDrop add up to a total of $300. The assembly takes in average 5 h and it requires previous experience in soldering surface-mount components; (b) Operation costs are hard to estimate because they are highly specific to the bio-application: the reagents for gene cloning cost $100, while fragrances cost less than $1. Our approach to reducing the volumes of operational fluids is to keep the electrode size, and thus the droplet size, minimal.

Accessibility is a key feature for ensuring a high replicability of our platform. In general, users have no access to the microfabrication facilities needed to produce a microfluidic chip. Moreover, the services provided by some of the few specialized companies can easily get too expensive as they charge for design, fabrication, transport and communication. We made the fabrication of OpenDrop highly accessible for users: our architecture is compatible with a widely spread fabrication technique: printing circuit boards. Thus, anyone can order an OpenDrop online from a generic PCB fabrication shop [25,26] at a fraction of the cost.

Moreover, we enabled customized fabrication by open-sourcing the OpenDrop design files under the Creative Commons Attribution-ShareAlike license [27].

At-home operation of microfluidic machines is not trivial. In our case, to move droplets, the electrodes of the biochip have to be coated with a thin hydrophobic layer. The common procedure is to mix the insulator and hydrophobic chemicals and then apply them using either spin coating followed by temperature curing (e.g., for Teflon) or vapor deposition (e.g., for Barium Strontium Titanite) [12,28]. To ensure OpenDrop is operable at home, we developed a coating technique based on thin film. It can be applied quickly, with no need for additional equipment or a clean room.

**Figure 3:**
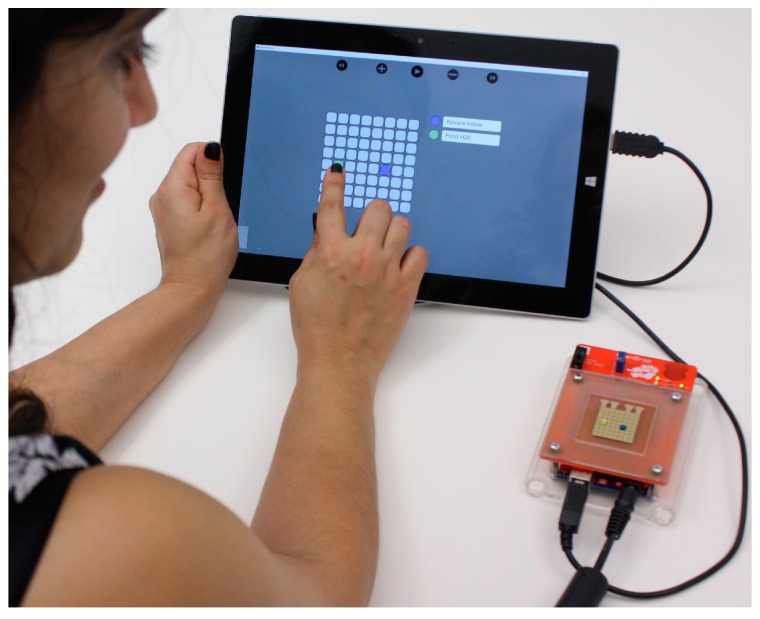
Our proposed system consists of an OpenDrop machine, a personal computer (tablet, smart phone) and the fluids needed for the target bio-application. The droplets are controlled real-time through the software interface by using touch-based gestures, such as drag and drop.

Lastly, we developed a software tool with a user-friendly interface that allows the user to interact real-time with the droplets using hand-gestures on a touch screen. In Figure 3, we show the user merging two droplets by dragging them to the same location. The software tool interprets the finger drag, computes the electrode actuation sequence and automatically moves the droplets accordingly.

## 4. Experimental Setup

Figure 1 presents OpenDrop, our cheap DIY biochip that can be operated at home by users using a simplified interaction technique based on touch. A wetlab in miniature, OpenDrop uses micro-droplets to execute bio-applications (“bio-protocols”).

As shown in Figure 3, the system setup we propose consists of an OpenDrop machine, a personal computer and the fluids needed for the target bio-application. After loading the OpenDrop reservoirs with fluids, the user programs the movement of the droplets through the software that runs on the computer. We designed the software to run through the internet browser, thus it is highly portable and can also be run on laptops, tablets or even smart phones.

At system level, our proposed setup is minimal and ideal for home usage. As presented in Section 6, we further propose several techniques to overcome the expertise needed for microfluidics. In the next paragraphs, we present the experimental setup needed for microfluidics, emphasizing the difficulties that users encounter.

**Figure 4:**
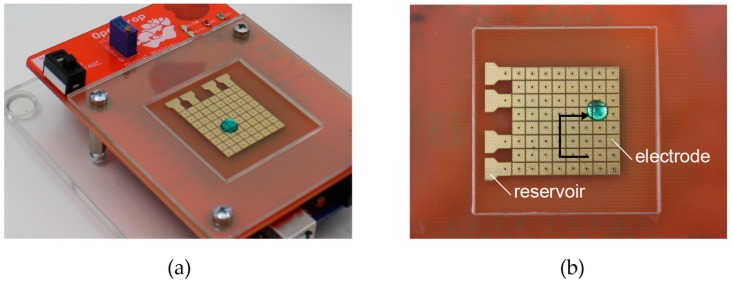
(**a**) OpenDrop has an array of 8 × 8 electrodes with four reservoirs; (**b**) The electrodes are reconfigurable and thus they can be used interchangeably.

As shown in Figure 4, OpenDrop consists of an array of electrodes, with each electrode capable of holding a droplet. The electrodes are coated with a 3–10 µm layer, with insulating and hydrophobic properties. Thus the droplets are not in direct contact with the electrodes.

Depending on the properties of the droplet, a filler fluid, such as oil, may be required. Although more difficult to achieve, it is preferred to move droplets in air, as oils can interfere with biological samples.

**Figure 5:**
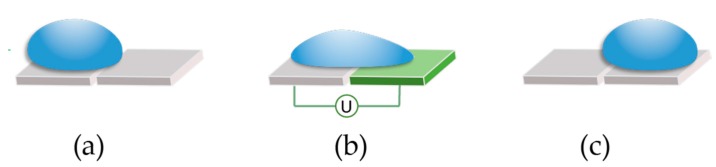
EWoD explained. (**a**) The usual shape of a droplet in the absence of voltage. The electrode underneath is coated with a hydrophobic layer, thus the droplet does not wet the surface; (**b**) Electrical voltage unbalances the force equilibrium at the solid-liquid-vapor interface, causing the droplet to wet the surface; (**c**) Consequently, the droplet moves towards the charged electrode.

As mentioned, OpenDrop uses electrical voltage to turn ‘on’ the electrodes and thus, actuate the droplets. For example in Figure 5, if the electrode on which the droplet is resting, is turned off (Figure 5a), and the electrode on the right is activated by applying voltage (Figure 5b), the droplet will move to the right (Figure 5c). In order to be actuated, the droplet has to be large enough to overlap the gap between the neighboring electrodes. A droplet can move up, down, right, left and diagonally on the electrode array.

**Figure 6:**
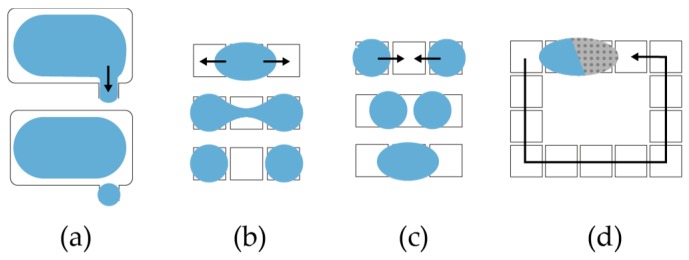
The microfluidic operations that can run on OpenDrop. The droplets are: (**a**) dispensed from reservoirs; (**b**) split; (**c**) merged and (**d**) mixed.

To dispense droplets from a reservoir, OpenDrop charges several electrodes to form a ‘finger’ droplet that eventually pinches into a child droplet (Figure 6a) [12]. To split a larger droplet into children droplets, OpenDrop charges concurrently two opposite electrodes to create opposite drag forces that eventually split the droplet (Figure 6b). To mix two droplets, OpenDrop first brings them together to merge (Figure 6c) and then transports them in a specific pattern (Figure 6d) [29,30,31].

The voltage values necessary to achieve EWoD depend on the targeted fluid, specifically on the surface tension at the interface between the fluid and the filler medium. Some samples can be impacted negatively by high voltage. In those cases, it is important to have a biochip that can actuate droplets at low voltage. Otherwise, using higher voltage is preferred because it increases the speed of droplets. We equipped OpenDrop with an adjustable power supply that can output between 50–260 V DC. Aqueous droplets can be moved with OpenDrop using 110 V. Similar results have been reported for previous EWoD-based biochips [11].

Our software interprets the drag gestures of the user and decides on the ‘electrode actuation sequence’ that specifies for each time step which electrodes have to turned off and on, in order to run the target bio-application.

## 5. Materials and Methods

**Figure 7:**
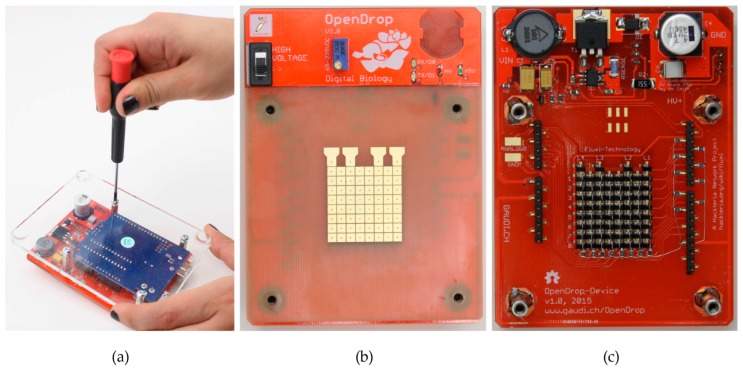
(**a**) We designed OpenDrop as a modular device that can be easily dis-assembled; (**b**) The front side of the PCB contains the electrode array and the high-voltage switch and regulator; (**c**) The back side of the PCB embeds the control circuit and the step-up voltage converter.

We designed OpenDrop for easy DIY fabrication. Figure 7a shows OpenDrop as a modular machine compatible with the Arduino, an accessible microcontroller currently having more than 400,000 users [32]. We used a double layer PCB, with electrodes on one side (Figure 7b) and the power supply and control on the other side (Figure 7c). The electrodes are gold-coated and connected through-hole to the control circuit.

**Figure 8:**
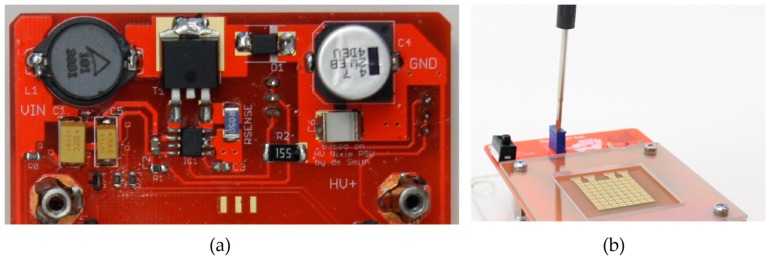
(**a**) Integrated power supply that steps up the voltage from 12 V to 260 V DC; (**b**) The user adjusts the voltage to fit the EWoD requirements for the targeted fluid.

To satisfy the voltage requirements for electromicrofluidics, we integrated a DC to DC power converter that steps up the voltage from 12 V to 260 V. The power converter was initially designed for Nixie tubes [33] and we adapted it to allow an adjustable voltage between 50 and 260 V DC (see Figure 8a). Thus, OpenDrop has integrated high-voltage control and can be operated directly from the common wall socket. The user has the option to adjust the voltage according to the type of fluid used by simply rotating the potentiometer using a screwdriver, as shown in Figure 8b.

**Figure 9:**
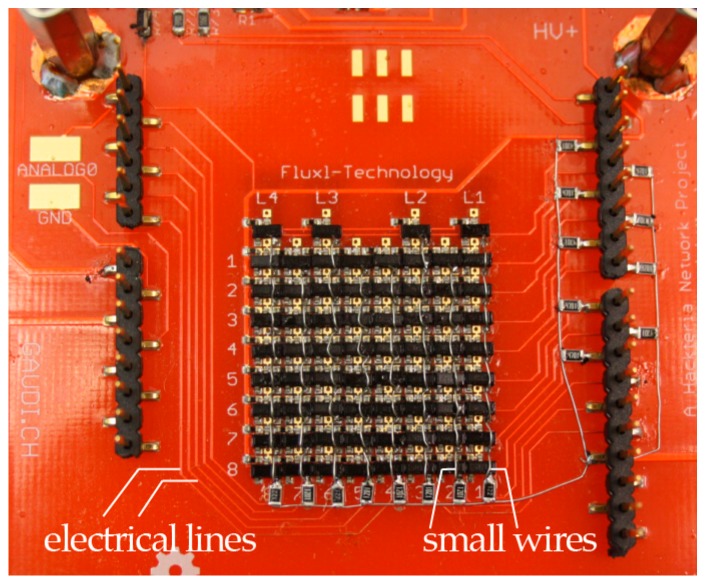
High Voltage Transistors used: BSS131, Infineon SIPMOS, Logic-Level, PG-SOT-23. Each transistor is connected to the corresponding electrode through a small through-hole wire, soldered manually. Electrical lines are transporting the high voltage from the power converter to the electrodes.

Electrical lines transport the high voltage from the power converter to the electrodes, as shown in Figure 9. The electrodes are switched on and off by high-voltage surface-mount transistors (BSS131, Infineon SIPMOS, Logic-Level, PG-SOT-23). To keep OpenDrop as a two-layer PCB (thus much cheaper than a multi-layer version) we connected the source pins of the transistors to the electrodes through small wires, see Figure 9.

For the coating, we acquired the materials as follows: Parafilm from Scienova GmbH [34], ETFE thin film 13 µm from Fluoroplasts GmbH [35], RainX from Conrad Electronic [36], Silicone oil 5 cst from QUAX GmbH [37], Cytop and Fluoropel from Cytonix LLC [38].

## 6. Interaction Techniques

The user experiences two major types of interactions with the OpenDrop system: at fluidic level and at software level.

### 6.1. Interaction with Fluids

At fluidic level, the user has to prepare the surface of the electrode array for electrowetting by applying a uniform coating. Considering the voltage limit of OpenDrop (250 V DC), the droplet must have a minimum contact angle of 120°, ideally 180° to be actuated [15]. Moreover, the coating has to be as thin as possible (in the range of 1–10 µm), and have a dielectric constant of 200–300 [12]. All these values are determined for the case when we move the fluids in air. Moving fluids in oil is less challenging in terms of coating. The goal of our work is to move the droplets in air.

#### 6.1.1. Adapting the Laboratory Coating Procedure

The traditional laboratory procedure consists of spin coating Teflon AF 1600, DuPont, 200-nm thick, or using vapor deposition of a mix of Parylene C and Cytop, then baking at 170° for 30 min [28]. We adapted this procedure in the context of running experiments on OpenDrop at home.

Since Teflon exceeded the targeted budget ($10/run), we used Fluoropel and spin-coated it over ITO glass [39]. A spin-coater is a relatively expensive machine (2000 to 5000 USD) that uses high-speed rotation to spin and spread a droplet of coating material into a thin layer. The viscosity of the material, the frequency and duration of the spinning determine the final thickness of the coating layer. The DIY version of a spin coater can be built for a fraction of the price [40].

Our DIY spin-coating technique worked reliably, but it has the following disadvantages:

- It requires a total of 4 h of work and a clean space (specks of dust can alter the quality of the coating and thus prevent electrowetting).
- It requires additional equipment such as a spin coater and an oven.
- The coating wears off in 24 h.

#### 6.1.2. Special Coating Technique at-Home Operation

We investigated coating techniques that are more suitable for users. We looked for techniques that can be applied fast (in less than 10 min), on the go (portable) and are accessible (using materials that can be acquired in a shop, not requiring authorization for specialized chemicals).

In the following paragraphs, we present such three different techniques.

Saran wrap and RainX are two products highly available on the market. Saran wrap, also known as cling wrap has a thickness of 10 µm and it constitutes a good insulator and dielectric material. RainX [36] is a product developed for car drivers to coat the windscreens against rain. We first tense the Saran wrap over a frame until there are no wrinkles left. Then we coat the wrap with RainX as instructed on the label (pour a generous amount, wait for one minute, rinse out with cold running water). We cut the wrap to the desired size, tape it on the OpenDrop carrier and apply it on the electrodes. To ensure a better adherence to the electrode surface, we spread a thin layer of kitchen oil on the electrodes, before applying the wrap. This coating does not last more than 2 h, and it needs access to a tap or running water.

Parafilm and Silicone oil are also easily accessible for users through online shops. Parafilm is an extensible film used in biological laboratories to seal Petri dishes. Similarly to the Saran wrap, we tense the parafilm to the maximum and apply it directly on the surface of the electrodes. We coat the top of the electrodes with a thin layer of silicone oil of viscosity cst 5.

**Figure 10:**
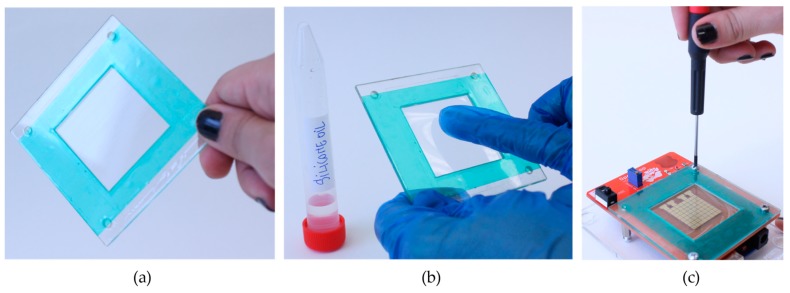
The simplest coating technique uses ETFE film and silicone oil. (**a**) The first step is to glue the ETFE film on the cartridge using double sided tape; (**b**) Next, the user applies a thin layer of silicone oil; (**c**) Finally, the cartridge is mounted on top of OpenDrop.

ETFE film and Silicone oil. The ETFE thin film is not stretchable and comes in different thicknesses. We experimented with films from various companies and found that ETFE 13 µm works the best. As shown in Figure 10b, a thin layer of silicone oil cst 5 is manually applied on top of the ETFE foil [41]. If the exposure to dust is minimized, this coating can last up to two-three days. For that, we recommend storing the coated ETFE film in a petri dish, or directly on the device, but covered with a glass top.

As mentioned, we envisioned our system to be used at home by everyone, so we assumed the user is not in possession of a specialized goniometer to measure the contact angle. Alternatively, we propose the experimental approach: after coating, the user turns on the device and checks whether the droplet moves or not. Our findings show that, without any theoretical knowledge about the contact angle, the users develop an intuitive feeling on whether the coating is hydrophobic enough to actuate the droplets. Thus, after several days working with OpenDrop, the users were able to visually inspect the droplet and estimate the contact angle.

### 6.2. Interaction with the Software

**Figure 11:**
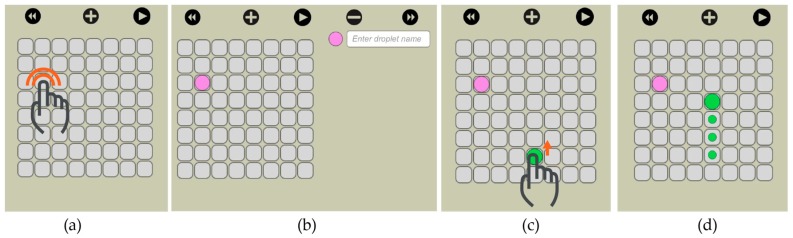
Through the software the user interacts with the droplets. (**a**) A new droplet is created by double tap on an empty electrode; (**b**) The user has the option to give a descriptive name to the droplet; (**c**) When using finger drag, the user decides on the route of the droplet that can be backtracked; (**d**) by looking at the traces.

Our visual interface allows the user to select the size of the electrode array and then visually actuate the droplets to the desired locations. We implemented this feature through drag and drop. On the virtual electrode array, the user creates a new droplet (double click) and then drags it to the desired location (Figure 11).

**Figure 12:**
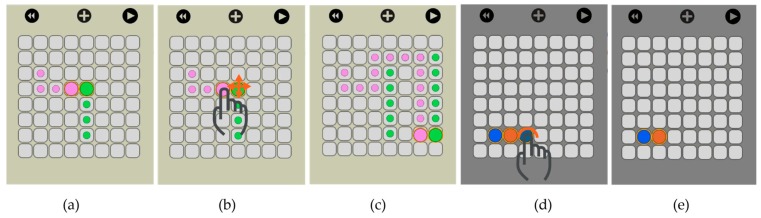
(**a**) When two droplets meet, they instantly merge together and (**b**) the user can manipulate them as a single entity using finger drag; (**c**) The merged droplets follow the drag as a unit; (**d**) In delete mode, with a single tap, the user can (**e**) delete droplets.

The user has the option to simulate the movement of the droplets before actually executing it on OpenDrop. As shown in Figure 12, traces are visible at any time to indicate the droplet routes and allow the user to visually predict an eventual undesired merge even before simulation. When two droplets meet, either vertically, horizontally or diagonally, they merge into a larger droplet that can be split afterwards by using two-finger drag gesture.

## 7. Results

The goal of our work is to investigate whether users can fabricate and use a biochip. As presented in the previous sections, we made OpenDrop accessible through design and operability techniques. In the next paragraphs we present our results obtained in two years after OpenDrop was released.

### 7.1. Replicability Study

To make OpenDrop accessible to as many people as possible, we used the following strategy:

1. We provided the design files as open source resources on github [42] and personal websites [43]. That way, anyone has the possibility to download, customize and make their own biochips.
2. We made a significant effort to document the fabrication, operation and various applications of OpenDrop. Proper documentation is a key element to ensure replicability. We maintained the documentation periodically. Apart from written documentation, we posted video tutorials on accessible channels, such as YouTube and Vimeo.
3. We initiated and developed a community of users by offering them technical support when needed. We implemented this step by hosting forums and organizing international meetings. We emphasized the educational aspect by giving lectures, course and weekly online seminars.

**Figure 13:**
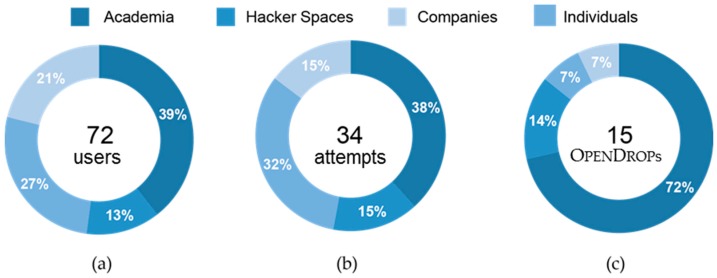
Our user base has 72 members, out of which 34 have tried to replicate OpenDrop. A total of 15 users (47%) succeeded to replicate our platform. We show the distribution of users by occupation for (**a**) the total user base; (**b**) the number of users that attempted to replicate OpenDrop; and (**c**) the users that succeeded to replicate OpenDrop.

Figure 13 shows the distribution of our user base over different occupational sectors. We are aware of 100 users that have expressed their interest in owning an OpenDrop, out of which 72 users are in regular contact forming our total user base (39% academia, 27% companies, 21% hacker spaces and 13% individuals). Almost half of our total user base, that is 34 users, tried to replicate OpenDrop (Figure 13b). Although both industry and academia showed a similar interest in replicating our platform, the users from academia were by far the most successful (Figure 13c).

Geographically, most interest was concentrated in North America and Europe, but lately a few Asian and South American research labs have expressed their interest in replicating and using an OpenDrop.

As mentioned, we observed the biggest interest in the academic sector. Researchers from technical domains (such as computer science or electrical engineering) used OpenDrop as an easy and cheap way to develop customized bio-applications [44,45,46]. Larger companies, such as Autocad and Novozymes, as well as start-ups [47,48] were also interested in replicating OpenDrop. A limited amount of OpenDrops found their home in various hacker spaces [49,50] or with individuals [51].

The interest of the users varied from using OpenDrop for educational purposes to adapting and customizing the design for specific applications. DropIO project from the Tangible Media Lab [45], MIT adapted OpenDrop to have a much larger array of electrodes. Their purpose is to have a microfluidic-based display that allows the study of various interaction techniques. The Computer Architecture Group from Bremen [44] increased the modularity of OpenDrop to support several layers that can be stacked on top of each other. Trojok et al. developed an adaptor that makes OpenDrop compatible with paper microfluidics [52].

### 7.2. Bio-Applications

Developing a new bio-application in general takes about 1.5 years of research. Thus our users are just in the incipient stage of bio-application development.

Currently, we are aware of work-in-progress for several applications as follows.

Tissue printing: Using OpenDrop to move and arrange droplets containing cells. Similar to the recent work of Chiang et al. [53], researchers at the Center for Regenerative Medicine Munich, Germany are working on integrating OpenDrop with a DIY bioprinter they built by modifying a 3D printer. While the bioprinter can arrange the cells in space, it cannot determine a temporal arrangement as the cells are printed sequentially. OpenDrop can overcome this issue by actuating cells in microgels in parallel and at programmed time intervals. Thus the cells will arrive at their specific location at the right time to communicate with each other and form a tissue.

Synthetic biology: Using OpenDrop to transform bacteria cells. Recently, researchers have proven that *E. coli* can be transformed on an electromicrofluidic platform by using magnetic beads [54]. We are aware of several research laboratories that are pushing the boundaries further as in trying to adapt the existing DIY kits for CRISPR/CAS9. The kit developed by Odeon for home use is an ideal candidate for OpenDrop. If successful, this project will enable users to genetically transform organisms in a much more accessible way, through an automatic touch-based interactive software.

Phage therapy: Using OpenDrop to generate new phages by altering genetically existing phages. As the antibiotic crises is becoming more and more life-threatening, phages seem the best solution so far for critical infections. However, phages cannot be administrated the same way at antibiotics, based on ‘one size fits all’ principle. Phages need to be administrated in a personalized cocktail for the specific patient and they cannot be stored for long time. This project [47] aims at using OpenDrop as a computational machine to explore the solution space of new phage cocktails. The envisioned scenario is the following: in case of infection, the user isolates the targeted bacteria, then loads OpenDrop with the closest phage known so far for the targeted bacteria. OpenDrop iteratively inserts mutations in the phage to design new organisms. The new phages are placed with the targeted bacteria and their action is monitored. In case of success, the user orders the phage cocktail from a synthesis company and takes it as a cure. From a broader perspective, the success of this project is a first step towards enabling everyone to ‘print’ their own medical cure.

Interactive displays: Using OpenDrop to mix colors in a controlled and precise range. Electrowetting on dielectric (the physics phenomenon that actuates the droplets on OpenDrop) was used for screens and displays. Thus, coming back, 50 years later, to the same area of application is not a surprise. Currently, researchers in human computer interaction [45] are interested in studying various ways of interacting with a display in a real-time manner. For that purpose, they have extended OpenDrop to a much large array of electrodes, in the range of thousands. Every droplet becomes a pixel that can be manipulated in real-time by the user to have the desired position and color.

Perfume making: Using OpenDrop to design new fragrances for real-time scenarios. Fragrance design is still a cumbersome manual task executed by specialized perfumers. OpenDrop could explore the perfume design space in a much faster and automated way, making it possible for users to design personalized perfumes. With additional equipment that connects the droplet outlets to the user’s nose, OpenDrop can also be used for augmented reality. In this scenario, the olfactory sense of the user is augmented by adding a programmed real-time fragrance.

Currently, OpenDrop is limited to bio-applications based on sequences of dispensing, splitting, merging, and mixing droplets. With the aid of various sensors (in the future, integrated) and additional equipment (e.g., temperature and magnetic bars, spectrophotometer), OpenDrop will be able to monitor, incubate and separate (chemically) the fluids.

## 8. Related Work

The only comparable work so far is the Dropbot [19], developed at Toronto University, Canada. In Table 1 we draw a schematic comparison between the major features of the two devices.

**Figure 14:**
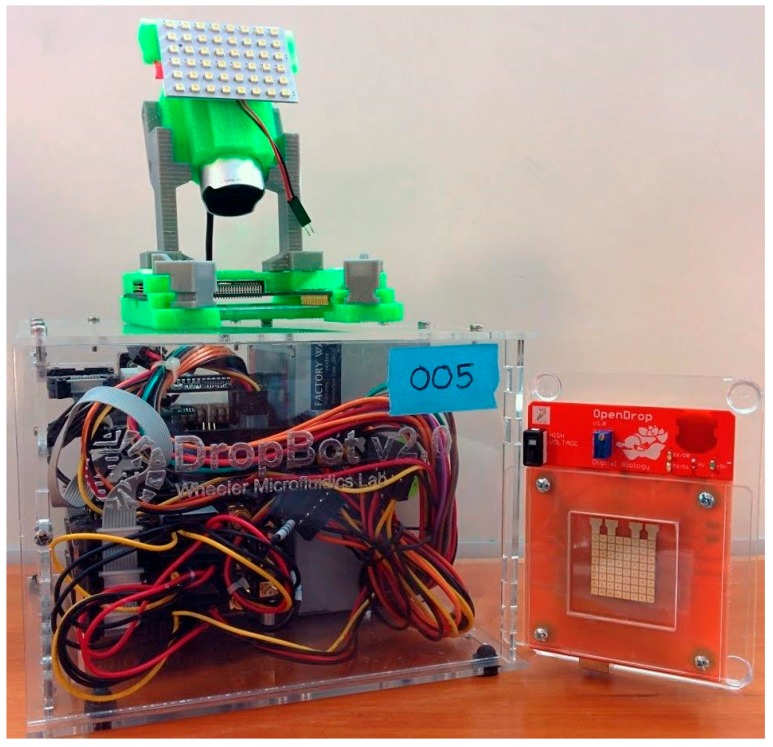
Dropbot (**left**) and OpenDrop (**right**) picture by Ryan Fobel.

Dropbot uses a chromium substrate for electrodes, fabricated by vapor-deposition. These electrodes are much more reliable than the PCB version, however, they cost 50% more than the cost of fabricating the entire OpenDrop. To actuate droplets, Dropbot uses AC voltage with an additional power supply at much larger size than the machine itself. In terms of coating, Dropbot uses an advanced nanocoating, at a high price range ($500) suitable for academic laboratories. In Figure 14, we show a side-by-side comparison of the Dropbot and OpenDrop.

In a more centralized way than our approach, the Dropbot design files were released open source and a forum run by the Dropbot authors was setup for common discussions and support. This approach ensured the quality of the information and support. Since our targeted users could basically be anyone, we chose an approach that would maximize the spread of our platform. In a de-centralized manner, we encouraged anyone to post the OpenDrop design files on their own websites and also host a discussion around its use.

Generally, Dropbot wins in terms of reliability and robustness, but pays the price of costs that cannot be afforded by makers or technology enthusiasts. Recently, we have established a collaboration with Fobel et al., together working on a hybrid platform that combines the advantages from both Dropbot and OpenDrop.

## 9. The Next Generations of OpenDrop

**Figure 15:**
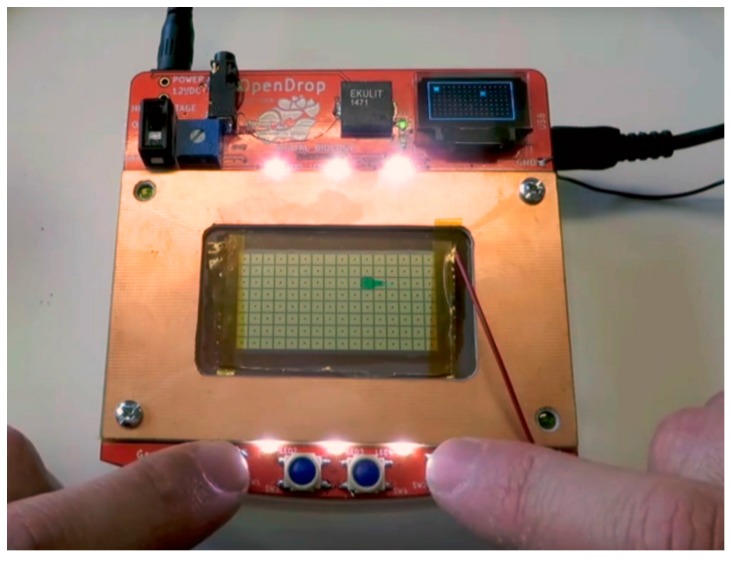
The front side of the next generation OpenDrop. The four buttons allow direct manipulation of droplets. The display on the top right side shows real-time the droplet movement.

Thus, OpenDrop is in continuous transformation, with new versions being released every six months. In the current version 2.1, showed in Figure 15, OpenDrop has the following features: much larger array of electrodes, four buttons for direct manipulation of droplets and a display that shows the exact location of droplets.

**Figure 16:**
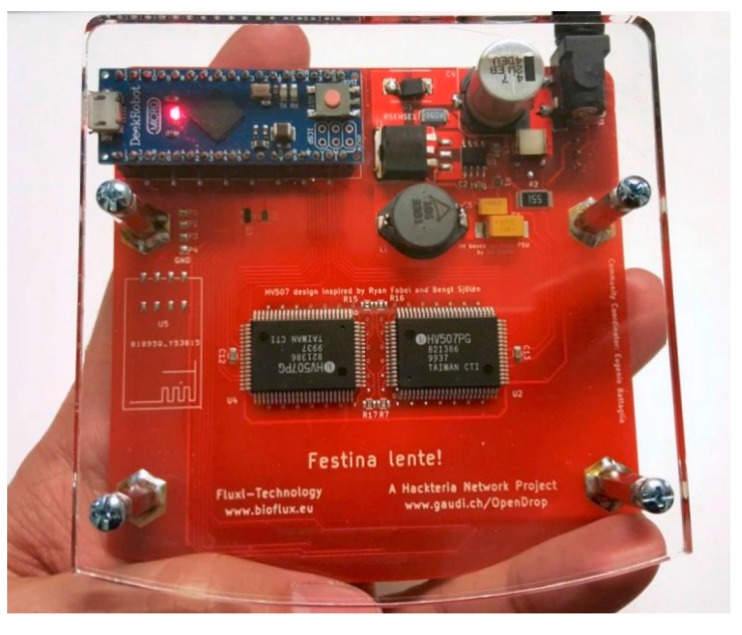
The back side of the next generation OpenDrop. We used a smaller Arduino and replaced the transistor array with two 64-Channel serial to parallel converters for high voltage.

The control circuit was also modified to adapt to the larger array of electrodes. As shown in Figure 16, the transistors were replaced with the high-voltage chips HV507.

The next generations of OpenDrop are a product of the fruitful collaboration inside the community we built. Thus, newer and better OpenDrop versions are being released every three-four months by users. In some cases, we are aware of their work and support it with technical knowledge, but there have also been several unexpected developments of OpenDrop.

## 10. Discussion and Dreams

In this paper, we presented OpenDrop, our electromicrofluidic platform for personal use. Our goal was to explore whether interested users, regardless of their level of expertise, can fabricate, own and use an electromicrofluidic platform. For that purpose, OpenDrop was designed to be cheap, DIY and accessible. We also implemented a software tool with visual interface and simple interaction. The tool automatically routes the droplets based on the drag-and-drop input from the user. OpenDrop can be operated remotely from a computer or a smart phone.

We have tested the replicability of the OpenDrop platform over two years by releasing the design files and building a community sharing platform. In terms of usability, the users managed to operate their platforms at home and are working on designing their own bio-applications.

Our findings show that 47% of attempts to replicate it were successful. We learnt that the main challenge in replicating OpenDrop is the hardware assembly phase. Since we targeted *everyone*, we got many users that have very limited knowledge of electrical engineering. For them, the bottleneck was soldering and testing for defects. We encouraged these users, most of which had expertise in biology or other fields, to team up with users that had complementary skills.

The main concerns are around an ethical and legal use of our platform. We have carefully considered the implications of giving everyone access to a machine that has the potential of a full laboratory. Thus, we have run a systematic educational program with the users that expressed interest in OpenDrop. Our program consisted of full courses, lectures, workshops and weekly online webinars. The purpose of our educational series is to cultivate a sense of responsibility in the users’ conduct. Prior to that, we had several meetings with bio-ethicists, scientific consultants and policy makers in Germany, to better understand their concerns regarding our work. We have included all of their advice and feedback in our educational platform.

## Figures and Tables

**Table 1:** Comparison between Dropbot and OpenDrop.

	Dropbot	OpenDrop
Electrode substrate	Chromium (vapor deposition, $500)	Golden PCB (etching, $10)
Coating technique	Nanocoating	Thin film and oil
Power supply	AC	DC
Community	Centralized, academic	Decentralized, makers
